# Gastrointestinal bleeding care across hospital and EMS settings in the Almaty region: a cross-sectional survey of organization, practice, and service capacity

**DOI:** 10.3389/frhs.2026.1918526

**Published:** 2026-08-18

**Authors:** Sungat Otegen, Mohammad Khoshraftar, Lyudmila S. Yermukhanova, Abylai Baimakhanov/Baimahanov, Aruzhan Otegenova Muratkyzy, Altynay Abdu Muratqyzy, Murat Jakanov, Aizat Seidakhmetova, Alireza Afshar

**Affiliations:** 1 Department of Public Health and Public Health Care, West-Kazakhstan Marat Ospanov Medical University, Aktobe, Kazakhstan; 2 Student Research Committee, Bushehr University of Medical Sciences, Bushehr, Iran; 3 Dean of the Faculty of Postgraduate Education of Asfendiyarov Kazakh National Medical University, Almaty, Kazakhstan; 4 Department of General Medicine, Asfendiyarov Kazakh National Medical University, Almaty, Kazakhstan; 5 Department of Therapy, Asfendiyarov Kazakh National Medical University, Almaty, Kazakhstan; 6 The Head of the Department of General Surgery, West Kazakhstan Medical University Named After Marat Ospanov, Aktobe, Kazakhstan; 7 Head of the Department of Emergency Medical Care and Nursing, Dean of the Faculty of Medicine, South Kazakhstan Medical Academy, Shymkent, Kazakhstan

**Keywords:** emergency medical services, emergency service, gastrointestinal hemorrhage, hospital, physicians' practice patterns, time factors

## Abstract

**Background:**

Gastrointestinal bleeding (GIB), particularly upper gastrointestinal bleeding (UGIB), requires rapid coordination between emergency medical services (EMS) and hospital teams, yet evidence on how service organization affects timeliness and practice is limited in Kazakhstan.

**Aim:**

To evaluate the organization of GIB care in Almaty region, with emphasis on clinician knowledge and practice, EMS performance, and organizational features of hospital care.

**Methods:**

We conducted a cross-sectional survey between March and October 2024 involving two broad respondent groups and three distinct samples: a hospital-based group comprising 99 inpatient clinicians and 99 hospital representatives, and an EMS group comprising 120 personnel. The three samples completed different questionnaires and were analyzed separately. Descriptive analyses, chi-square or Fisher's exact tests, and exploratory Spearman correlations were used.

**Results:**

Inpatient clinicians showed near-universal agreement with guideline-based UGIB management principles, but self-reported adherence was inconsistent. Only 1.0% reported always using validated risk scores, and 99.0% reported only sometimes protecting the airway before endoscopy in patients with active hematemesis and altered mental status. In unadjusted respondent-level analyses, clinicians with ≥5 years of experience more frequently reported adequate training and perceived risk scores as useful (both *p* < 0.001). In the EMS group, all six respondents reporting arrival within 10 min were in the ≥16-team category (6/58, 10.3%; Fisher's exact *p* = 0.047). Reported ambulance-team availability showed a modest inverse correlation with continuous arrival time (*ρ*=−0.277, *p* = 0.002), while completion of selected examinations and vital-sign assessments differed across team-availability categories (all *p* < 0.001). All hospital representatives reported 24-hour endoscopist availability, but important elements of written protocol content were reported less consistently.

**Conclusions:**

UGIB care in the Almaty region showed consistently described structural endoscopy availability but incomplete protocol content, together with selected inpatient-practice and prehospital differences. The EMS findings were exploratory and require confirmation using operational and patient-level data.

## 1 Introduction

Upper gastrointestinal bleeding (UGIB) is a global emergency with incidence ranging from 47 to 172 per 100 000 persons and more than 300 000 U.S. admissions annually (1, 2). Despite advances in Helicobacter pylori eradication, proton-pump inhibitors and endoscopic therapy, case-fatality rates remain 2%–10% (2, 3), reflecting an older, comorbid population frequently using non-steroidal anti-inflammatory drugs and anticoagulants (2). UGIB arises proximal to the ligament of Treitz; non-variceal causes like peptic ulcer disease, gastritis, Mallory-Weiss tears, vascular malformations and malignancy predominate, whereas variceal bleeding from portal hypertension carries higher mortality (3, 4). Risk factors include Helicobacter pylori infection, NSAIDs, low-dose aspirin, selective serotonin-reuptake inhibitors and anticoagulants (2, 4). Hospitalized patients are predominantly male with a mean age around 52 years (2); economic costs exceed USD 7.6 billion (1). Management requires rapid resuscitation with airway protection, crystalloid fluids, restrictive transfusion thresholds of 7–8 g dL⁻^1^ and vasoactive therapy such as octreotide for suspected variceal bleeding (3, 5, 6).

International guidelines recommend using validated risk scores to triage UGIB patients and performing esophagogastroduodenoscopy within 24 h after stabilization (7–9). Observational data show that endoscopy identifies the source in roughly 80% of cases (3) and that urgent procedures (< 6–12 h) do not consistently reduce mortality and may increase rebleeding (10, 11). Implementation of risk scores remains inconsistent (12), and prehospital resources vary widely: only 12% of suspected UGIB patients receive prehospital interventions (13) and few facilities offer 24-hour endoscopy (14). Prehospital care emphasizes basic resuscitation (9), and high-volume centers report > 90% success rates for endoscopic hemostasis (15).

Most research and guidelines center on in-hospital care, leaving prehospital management relatively unexplored. Emergency medical services (EMS) providers face diagnostic uncertainty, limited therapeutic options and variable access to specialist consultation. A narrative review for prehospital clinicians emphasizes simple measures: maintaining airway patency, administering supplemental oxygen, positioning the patient appropriately, frequent suctioning, giving antiemetics and avoiding sedating drugs. Peripheral intravenous or intraosseous access should be established promptly, and crystalloid fluids infused to maintain perfusion (3, 9). However, few EMS agencies carry blood products or have point-of-care tests, and prehospital risk scores are rarely applied (9, 16). A prospective French study found that only 12% of patients with suspected UGIB received prehospital medical intervention and that the incidence of UGIB requiring EMS response was approximately 122 per 100 000 person-years (13). Helicopter emergency service reports from Japan describe improved vital signs after air evacuation but highlight the limited number of facilities with 24-hour endoscopy and the need for prompt decision-making in resource-limited settings (14). These observations illustrate the heterogeneity of prehospital care and its potential impact on timeliness and outcomes.

Comparing the organization of care between prehospital and hospital settings reveals critical differences. In hospitals, multidisciplinary teams including emergency representatives, gastroenterologists, intensivists, surgeons, interventional radiologists and nurses work under written protocols, have access to risk stratification tools and can perform emergent EGD and radiologic interventions (9, 15). Success rates for endoscopic hemostasis exceed 90% in experienced centers (15). By contrast, EMS systems vary widely in staffing and infrastructure; some operate with a handful of ambulance crews, whereas others manage large fleets. Response times and adherence to recommended assessments may therefore depend on the number of available teams and the presence of specialized personnel. These differences justify evaluating organizational and clinical-practice features across both hospital and prehospital settings.

Regional context can also influence practice. In Central Asian countries like Kazakhstan, epidemiological data on UGIB are scarce. A retrospective study from West Kazakhstan reported that duodenal ulcer was the underlying cause of ulcerative gastroduodenal bleeding in 54%–62% of patients and that most patients were over 50 years old (17). Women constituted 57.7% of cases and active bleeding at endoscopy was uncommon. National statistics indicate that gastrointestinal bleeding is a leading cause of surgical admission and that non-variceal bleeding accounts for an estimated 8 000-9 000 cases annually, with mortality around 3%–4%. Rural regions face challenges such as long transport distances, limited access to endoscopy and variability in EMS staffing, which may delay definitive care (17, 18). To date, no published study has systematically compared the knowledge, attitudes and practices of inpatient clinicians and EMS providers or assessed how organizational factors (e.g., number of ambulance teams, existence of written protocols) influence timeliness of UGIB care in Kazakhstan.

Recognizing these gaps, the present cross-sectional study was designed to evaluate the organization of UGIB care in Almaty region, Kazakhstan. We aimed to assess clinicians' familiarity with evidence-based recommendations, examine self-reported adherence to best practices in both hospital and prehospital environments, and explore whether reported ambulance-team availability was associated with arrival time and selected clinical-assessment actions. By identifying deficiencies in early management and structural weaknesses, this work seeks to identify potential gaps and generate priorities for facility-level audit and prospective evaluation for patients with gastrointestinal bleeding.

## 2 Methods

### 2.1 Study design and setting

We conducted a cross-sectional observational study between March and October 2024 to evaluate the organization of care for gastrointestinal bleeding in Almaty region, Kazakhstan. The study involved two broad respondent groups and three distinct survey samples. The hospital-based group comprised 99 inpatient clinicians who completed the clinical knowledge and practice questionnaire and a separate sample of 99 hospital representatives who completed the organizational-readiness questionnaire. The prehospital group comprised 120 EMS personnel who completed the EMS questionnaire. The three samples completed different survey instruments and were analyzed separately. Participating hospitals included City Clinical Hospitals No. 4, 7, 8, and 12, while EMS respondents worked within the single municipal EMS system serving Almaty region.

### 2.2 Participants

Inpatient clinicians were eligible if they were board-certified surgeons, intensivists or endoscopists currently working in the participating hospitals, routinely engaged in the management of UGIB and willing to complete a self-administered questionnaire. EMS respondents were paramedics, nurses or representatives employed by the city emergency medical station who were involved in field assessment and transport of patients with suspected gastrointestinal bleeding. Hospital representatives were senior administrators, such as department heads or quality managers, who could provide information about institutional protocols and resource availability. Individuals not directly involved in UGIB care or with incomplete survey responses were excluded. Using convenience sampling, we obtained responses from two broad respondent groups comprising three distinct samples: 99 inpatient clinicians and 99 hospital representatives in the hospital-based group, and 120 EMS personnel in the prehospital group. Because the samples completed different questionnaires and served different analytic purposes, their results were analyzed and reported separately rather than combined into a single pooled sample.

### 2.3 Survey instruments

Three related but distinct questionnaires were used. The organizational questionnaire was adapted from the National Confidential Enquiry into Patient Outcome and Death report *Time to Get Control?*, which evaluated hospital-level structures and protocols for the management of severe gastrointestinal hemorrhage (19). Because the adapted questionnaire was administered to individual hospital-based representatives rather than completed once at each hospital, its findings were interpreted as respondent-reported organizational features and not as independently verified hospital-level measurements. Accordingly, these responses were not interpreted as measurements from 99 hospitals and were not independently verified at the four-facility level. The clinical-practice and EMS questionnaires were study-specific item sets developed by mapping questions to recommendations in contemporary UGIB guidelines and relevant emergency-care literature (7, 9, 16, 19, 20).

Before field administration, the draft questionnaires were reviewed by six members of the multidisciplinary research team. The review addressed clinical relevance, item clarity, contextual suitability, and consistency of the response options. The questionnaires were administered in Kazakh. The source items were translated into Kazakh and reviewed by bilingual members of the research team for semantic clarity, clinical accuracy, and contextual appropriateness. A formal cross-cultural validation study involving independent back-translation was not undertaken. Before the main survey administration, the questionnaires were informally pretested with members of the intended professional groups to identify unclear terminology, ambiguous questions, unsuitable response options, and practical difficulties with completion. Feedback was used to clarify selected terms, simplify item wording, and harmonize response options. This pretesting was intended to improve clarity and usability and was not designed as a formal psychometric validation study (21–23).

The questionnaires underwent multidisciplinary review and informal pretesting to improve clinical relevance, clarity, and contextual appropriateness; however, formal psychometric validation was not undertaken. Accordingly, the organizational instrument is described as an adapted questionnaire, while the clinical-practice and EMS instruments are described as study-specific questionnaires rather than validated measurement scales. Because the knowledge, attitude, and practice items represented distinct clinical beliefs and actions, they were analyzed individually rather than combined into composite scores. Where estimable, Cronbach's *α* was reported only as an indicator of internal consistency and was not interpreted as evidence of questionnaire validity (21, 22, 24).

Knowledge and attitude items (labelled Q7a-Q7i for inpatients) assessed agreement with core recommendations such as performing laboratory tests, applying validated risk scores, documenting orthostatic vital signs, placing large-bore intravenous catheters, administering crystalloid fluids and supplemental oxygen, admitting non-responders to monitored care and protecting the airway prior to endoscopy in patients with altered mental status. Responses were recorded on a 5-point Likert scale ranging from “strongly disagree” to “strongly agree.” For EMS personnel, knowledge questions focused on call volume, training adequacy and communication practices. Post-collection review of the administered questionnaire identified Q7d as a verbatim duplication of Q7c. Because Q7d did not represent a distinct content item and Q8d assessed a different practice, namely documentation of nasogastric aspirate findings, Q7d was excluded from retained-item and psychometric analyses, and Q8d was not included in the paired endorsement-implementation analysis (22).

Self-reported practice items (Q8a-Q8i) asked clinicians how often they ordered laboratory tests, applied risk scores, documented orthostatic vitals, recorded nasogastric aspirate findings, used large-bore intravenous catheters, infused crystalloids for hypovolemia, referred patients to monitored or intensive care units and protected the airway prior to endoscopy. Practice frequencies were recorded on a 5-point scale from “never” to “always.” EMS practice items evaluated operational characteristics (population served, number of ambulance teams, specialized team presence), average call volume for gastrointestinal bleeding and adherence to specific clinical actions such as performing chest and abdominal examinations, measuring pulse and blood pressure, providing follow-up communication with receiving hospitals and arrival time to the patient after receiving a call. Arrival time was reported in minutes and categorized (<10 min vs. ≥10 min) for analysis. The hospital-level survey asked whether written guidelines existed for non-variceal and variceal bleeding, whether they covered interventional radiology and surgical procedures, whether recommendations on discontinuing antithrombotic agents were included and whether 24-hour endoscopy and emergency response teams were available.

### 2.4 Data collection and management

Data collection was performed by trained research assistants who distributed printed questionnaires in person at staff meetings and EMS shifts. Completed surveys were returned anonymously in sealed envelopes. Responses were coded and entered into IBM SPSS Statistics (version 29, New York, USA). For each variable, coding reflected the directionality of adherence; higher numeric codes typically indicated more frequent adherence or stronger agreement. Questionnaire responses were retained in their original item-level categories, and coding direction was verified against the questionnaire and codebook before analysis. Because the knowledge, attitude, and practice items did not demonstrate sufficient internal consistency to support composite scoring, no summed or averaged score was retained. Each questionnaire item was analyzed separately. Surveys with missing demographic or outcome data exceeding 20% were excluded from analysis. During re-verification of the dataset, one EMS respondent was found to have entered an arrival time of 6 min while selecting ≥10 min on the separate categorical arrival-time item. Because the original questionnaire could not be used to adjudicate this discrepancy, the categorical and minute-valued variables were retained and analyzed as separately recorded.

### 2.5 Statistical analysis

All analyses were performed using IBM SPSS Statistics, version 29.0 (IBM Corp., Armonk, NY, USA). Descriptive statistics were used to summarize respondent characteristics and individual questionnaire items. Continuous variables were presented as means with standard deviations or medians with interquartile ranges, as appropriate, while categorical variables were presented as frequencies and percentages. Categorical associations were examined using Pearson's chi-square test when expected-cell assumptions were satisfied and Fisher's exact test when cells were sparse. Spearman's rank correlation was used for selected ordinal or non-normally distributed variables. Only item-level variables were included in these correlations; no composite practice score was used. Because the questionnaire items did not support construction of a reliable practice composite, all knowledge, attitude, and practice items were analyzed individually. No multivariable model was fitted for EMS arrival within 10 min because only six respondents reported this outcome. The individual respondent was the unit of analysis. Unique hospital and EMS operational-unit identifiers were not collected; therefore, within-cluster correlation could not be estimated and cluster-adjusted analyses could not be performed. Respondent-level *p* values are consequently unadjusted for clustering and should be interpreted as exploratory (25, 26). All analyses were considered exploratory, with statistical significance defined as *p* < 0.05.

### 2.6 Ethical considerations

The study protocol was reviewed and approved by the Institutional Review Board of West-Kazakhstan Marat Ospanov Medical University, Aktobe, Kazakhstan (approval number 11.23/03). Participation was voluntary, and all respondents provided written informed consent before completing the anonymous questionnaire. The questionnaires contained no personal identifiers. Data were stored on password-protected computers accessible only to the research team and were reported in aggregate to preserve confidentiality. The study adhered to the ethical principles of the Declaration of Helsinki.

## 3 Results

### 3.1 Internal consistency of questionnaires

Internal-consistency diagnostics did not support combining the inpatient questionnaire items into composite scales. After exclusion of the duplicated Q7d item, seven of the eight retained Q7 items had no response variability, precluding meaningful estimation of internal consistency for that block. The Q8 practice block had a raw Cronbach's *α* of −3.788 and a standardized *α* of 0.155, while the Q11 attitude/confidence block had a raw *α* of 0.057 and a standardized *α* of 0.136. Consequently, the Q7, Q8, and Q11 items were analyzed individually. Although the EMS clinical-action block had a raw *α* of 0.741, it was treated as a checklist of distinct actions rather than combined into a composite score (Supplementary Table S1).

### 3.2 Characteristics of respondents and facilities

Three respondent samples were included and analyzed separately: 99 inpatient clinicians, 99 hospital representatives, and 120 EMS personnel. The first two samples formed the hospital-based respondent group, whereas the third sample represented the prehospital EMS group. Table 1 summarizes the demographic and professional characteristics of the inpatient and EMS samples and the organizational information reported by the hospital representatives. The inpatient group was predominantly composed of men (92 of 99, 92.9%), almost all aged ≥40 years (91, 91.9%), with surgeons accounting for 55.6% of clinicians. Intensivists constituted 35.4%, while endoscopists made up 9.1%. A majority were permanent staff with <5 years' experience (65, 65.7%), and the remainder had ≥5 years' experience. UGIB was a common clinical encounter: two-thirds of clinicians reported managing at least three cases per week and one-third reported one case per week.

**Table 1:** Characteristics of inpatient clinicians and EMS personnel and organizational features reported by hospital-based respondents in the Almaty region, Kazakhstan.

Group	Variable	Category	Frequency (n)	Valid Percent (%)
Inpatient healthcare	Age group	<40	8	8.1
		≥40	91	91.9
	Sex	Female	7	7.1
		Male	92	92.9
	Specialty	Surgeon	55	55.6
		Intensivist	35	35.4
		Endoscopist	9	9.1
	Clinical position	Trainee	0	0
		Permanent <5 years	65	65.7
		Permanent ≥5 years	34	34.3
	Frequency of managing UGIB	Very often (≥3 patients/week)	65	65.7
		Often (1 patients/week)	34	34.3
		Sometimes/Rarely (<2 patients/month)	0	0
EMS respondents	Population covered by emergency	≤100k	66	55.0
		100–200k	13	10.8
		>200k	41	34.2
	Ambulance teams (number)	1–5	40	33.3
		6–15	22	18.3
		≥16	58	48.3
	Dedicated team for non-traumatic bleeding	No	120	100.0
		Yes	0	0
	Calls per shift for GIB	1–2 (few)	100	83.3
		3–4 (moderate)	20	16.7
		>4 (high)	0	0
	Arrival time after call	<10 min	6	5.0
		≥10 min	114	95
	Follow-up communication	No	8	6.7
		Yes	112	93.3
	Examination time	10–15 min	120	100.0
		<10 min	0	0
		>15 min	0	0
	Arrival time after receiving EMS call (minutes)	Mean ± STD	11.01 ± 2.62	
Hospital-based organizational respondents	Written guidelines for non-variceal UGIB	Yes	85	85.9
		No	14	14.1
	Guidelines include interventional radiology	Yes	51	51.5
		No	48	48.5
	Guidelines include surgical treatment	Yes	17	17.2
		No	82	82.8
	Guidelines include discontinuation of aspirin	Yes	17	17.2
		No	82	82.8
	Guidelines include discontinuation of clopidogrel	Yes	17	17.2
		No	82	82.8
	Variceal UGIB guidelines include antibiotic use	Yes	85	85.9
		No	14	14.1
	On-call schedule for endoscopy in the hospital	Yes	99	100.0
		No	0	0.0
	Endoscopist on-call coverage available 24 h/day	Yes	99	100.0
		No	0	0.0
	Emergency response team on site	Yes	99	100.0
		No	0	0.0

EMS respondents typically served populations of ≤100 000 (66 respondents, 55.0%) or >200 000 (41, 34.2%). Team capacity varied markedly: one-third of respondents operated 1–5 ambulance teams, 18.3% operated 6–15 teams and nearly half operated ≥16 teams. None of the respondents had a dedicated team for non-traumatic bleeding. Most shifts involved one to two calls for gastrointestinal bleeding (83.3%), while three to four calls were less common (16.7%). Only 5.0% of respondents reported arriving at the patient within 10 min of the call; the vast majority arrived after 10 min. Follow-up communication with receiving hospitals was reported by 93.3% of respondents, and the time spent examining the patient was consistently 10–15 min. The mean time from call receipt to patient arrival was 11.01 ± 2.62 min. These data are detailed in Table 1.

The organizational responses indicated consistently described structural endoscopy availability but less complete written protocol content. Most hospital representatives (85 of 99, 85.9%) reported written guidelines for non-variceal UGIB. Among these, interventional radiology or embolization procedures were explicitly covered in 51 respondents (51.5%), surgical treatment in 17 (17.2%) and recommendations on discontinuing aspirin or clopidogrel in 17 each (17.2%). Variceal bleeding guidelines often addressed antibiotic therapy (85 respondents, 85.9%). In addition, all 99 hospital representatives reported 24-hour endoscopy availability, an on-call endoscopy schedule and an emergency response team.

### 3.3 Inpatient knowledge, attitudes and associations with clinical position

As shown by the knowledge items in Supplementary Table S2, inpatient clinicians overwhelmingly endorsed core components of initial UGIB management. All respondents strongly agreed that the initial assessment should include laboratory testing (item 7a), use of validated risk scores (7b), placement of large-bore venous access (7e), crystalloid infusion for hypovolemia (7f), supplemental oxygen for hypoxemia (7 g), admission to monitored or intensive care for patients who did not respond to fluid resuscitation (7 h) and airway protection before endoscopy in cases of active hematemesis with altered mental status (7i). Documentation of orthostatic vitals in hemodynamically stable patients (7c) prompted a slightly wider spread of responses, with 64.6% choosing “strongly agree” and 35.4% choosing “agree”. Q7d was not interpreted as a separate item because the administered questionnaire duplicated Q7c verbatim; therefore, its distribution was retained for transparency but was not assigned a clinical interpretation or paired with Q8d.

Differences were observed when responses were stratified by clinical position. In Table 2, permanent staff with ≥5 years of experience more frequently reported sufficient education and training to manage UGIB than those with <5 years of experience (97.1% vs. 49.2%; Pearson *χ*^2^ = 22.65, *p* < 0.001). Clinicians with ≥5 years of experience also more frequently regarded UGIB risk scores as clinically useful (100.0% vs. 1.5%; Pearson *χ*^2^ = 94.69, *p* < 0.001). The corresponding two-sided Fisher exact *p* values were also <0.001. These respondent-level associations were unadjusted for potential institutional clustering and should be interpreted as exploratory rather than causal (25, 26).

**Table 2:** Association of clinical position with perceived training adequacy and perceived clinical usefulness of UGIB risk scores among inpatient clinicians.

Variables		Permanent position < 5 years (*n* = 65)	Permanent position ≥ 5 years (*n* = 34)	Total (*n* = 99)	Chi-square test		
					Pearson *χ*^2^	df	*P* value
Panel A. Perceived sufficiency of medical education and training	Agree	32 (49.2%)	33 (97.1%)	65 (65.7%)	22.65	1	<0.001
	Neither agree nor disagree	33 (50.8%)	1 (2.9%)	34 (34.3%)			
	Total	65 (100.0%)	34 (100.0%)	99 (100.0%)	Fisher's Exact Test (2-sided *p* < 0.001)		
Panel B. Perceived clinical usefulness of UGIB risk scores	No	64 (98.5%)	0 (0.0%)	64 (64.6%)	94.69	1	<0.001
	Yes	1 (1.5%)	34 (100%)	35 (35.4%)			
	Total	65 (100.0%)	34 (100.0%)	99 (100.0%)	Fisher's Exact Test (2-sided *p* < 0.001)		

Panel A: No respondents selected ‘Disagree' after recoding.

Panel B: Responses are presented using the recoded binary categories applied in this crosstab analysis (Yes/No).

Percentages were calculated within clinical-position categories. Pearson χ^2^ statistics and two-sided Fisher exact *p* values are both presented; Fisher exact testing was retained as an exact sensitivity analysis for the markedly imbalanced 2 × 2 distributions. All tests were conducted at the respondent level without adjustment for potential institutional clustering; therefore, the inferential findings should be interpreted as exploratory (26).

### 3.4 Self-reported practice patterns and paired endorsement-implementation findings in inpatient clinicians

Self-reported behavior did not mirror the uniformly high knowledge scores. Supplementary Table S3 presents the reported frequency of nine clinical actions in the initial management of UGIB. Ordering laboratory tests (item 8a) was most commonly reported as “always” (67.7%), with the remaining clinicians reporting “often”. Use of validated risk scores was not consistently reported as an “always” practice: one clinician (1.0%) selected “always,” 65 (65.7%) selected “often,” and 33 (33.3%) selected “sometimes.” Documentation of orthostatic vital signs (8c) was “always” for two-thirds of clinicians and “often” for the rest. All respondents reported documenting nasogastric aspiration findings and administering supplemental oxygen when indicated. Placement of large-bore intravenous catheters (8e) and crystalloid infusion for hypovolemia (8f) were reported as “often” by nearly all clinicians. ICU or monitored care admission for non-responders to fluid resuscitation (8 h) was also universally reported as “often”. Airway protection before endoscopy in patients with active hematemesis and altered mental status (8i) was reported as “sometimes” by almost all clinicians (99.0%), indicating that this action was not routinely reported in the specified clinical scenario.

In the inpatient group, clinical position was inversely correlated with the reported frequency of managing UGIB (*ρ*=−0.478, *p* < 0.001; Supplementary Table S4). This item-level correlation was exploratory and should not be interpreted as evidence that clinical position determines UGIB exposure.

The paired item-level analysis indicated that reported implementation discordance was concentrated in two practices. Although all 99 clinicians endorsed the use of a validated risk score, 33 (33.3%; 95% CI 24.8–43.1) reported using one less frequently than “often.” Similarly, all clinicians endorsed airway protection before endoscopy in the specified scenario of active hematemesis and altered mental status, but 98 (99.0%; 95% CI 94.5–99.8) reported implementing this intervention less frequently than “often.” Discordance was absent or uncommon for the remaining clearly matched actions (Table 3). Q7d was excluded because it duplicated Q7c in the administered Kazakh-language questionnaire. Consequently, Q8d was omitted from the paired endorsement–implementation analysis because it had no distinct corresponding Q7 endorsement item.

**Table 3:** Paired comparison of guideline endorsement and frequently reported implementation among inpatient clinicians (*n* = 99).

Matched clinical action	Endorsed recommendation, *n* (%)	Reported always/often, *n* (%)	Endorsed but performed less than often, *n* (%)	95% Wilson CI for discordance, %
Initial laboratory testing	99 (100.0)	99 (100.0)	0 (0.0)	0.0–3.7
Use of a validated UGIB risk score	99 (100.0)	66 (66.7)	33 (33.3)	24.8–43.1
Documentation of orthostatic vital signs	99 (100.0)	99 (100.0)	0 (0.0)	0.0–3.7
Placement of large-bore intravenous access	99 (100.0)	98 (99.0)	1 (1.0)	0.2–5.5
Crystalloid infusion for hypovolemia	99 (100.0)	99 (100.0)	0 (0.0)	0.0–3.7
Supplemental oxygen when indicated	99 (100.0)	99 (100.0)	0 (0.0)	0.0–3.7
Monitored or intensive-care admission for fluid non-response	99 (100.0)	99 (100.0)	0 (0.0)	0.0–3.7
Airway protection before endoscopy in the specified clinical scenario	99 (100.0)	1 (1.0)	98 (99.0)	94.5–99.8

Guideline endorsement was defined as “strongly agree” or “agree” on the corresponding Q7 item. Frequent implementation was defined as “always” or “often” on the corresponding Q8 item. Discordance was defined as endorsement of the recommendation accompanied by implementation less frequently than “often.” Q7d was excluded because it duplicated Q7c verbatim in the administered questionnaire. Consequently, Q8d had no distinct corresponding Q7 endorsement item and was omitted from the paired analysis. No composite score was constructed.

### 3.5 Emergency medical service performance and exploratory associations

Exploratory EMS analyses described how reported arrival time and selected clinical actions varied across categories of reported ambulance-team availability. Arrival within 10 min was reported only by respondents in the ≥16-team category (6/58, 10.3%), whereas no respondents in the 1–5-team or 6–15-team categories reported meeting this threshold (Fisher-Freeman-Halton exact *p* = .047). Because only six respondents were coded as arriving within 10 min, this comparison was treated as descriptive. The reported performance of three clinical-assessment actions also varied across ambulance-team categories. Examination of the chest and abdomen was reported by 7.5%, 31.8%, and 96.6% of respondents in the 1–5, 6–15, and ≥16-team categories, respectively [*χ*^2^(2) = 81.702, *p* < .001; Cramér's V = .825]. The corresponding Cramér's V values were.754 for pulse measurement and.870 for blood-pressure measurement (*p* < .001 for both; Table 4). These respondent-level associations were considered exploratory.

**Table 4:** Association of ambulance team availability with arrival time and selected EMS clinical actions for gastrointestinal bleeding (n=120).

Variables		1-5 teams (*n* = 40)	6-15 teams (*n* = 22)	≥16 teams (*n* = 58)	Total (*n* = 120)	Chi-square test			Cramér’s V
						Pearson χ²	df	*P* value	
Panel A. Arrival time after EMS call	<10 min	0 (0.0%)	0 (0.0%)	6 (10.3%)	6 (5.0%)	6.751	2	0.034	-
	≥10 min	40 (100.0%)	22 (100.0%)	52 (89.7%)	114 (95.0%)				
	Total	40 (100.0%)	22 (100.0%)	58 (100.0%)	120 (100.0%)	Fisher’s Exact Test (2-sided p=0.047)			
Panel B. EMS action item 1	No (0)	37 (92.5%)	15 (68.2%)	2 (3.4%)	54 (45.0%)	81.702	2	<0.001	0.825
	Yes (1)	3 (7.5%)	7 (31.8%)	56 (96.6%)	66 (55.0%)				
	Total	40 (100.0%)	22 (100.0%)	58 (100.0%)	120 (100.0%)	Fisher’s Exact Test (2-sided p<0.001)			
Panel C. EMS action item 2	No (0)	34 (85.0%)	15 (68.2%)	3 (5.2%)	52 (43.3%)	68.209	2	<0.001	0.754
	Yes (1)	6 (15.0%)	7 (31.8%)	55 (94.8%)	68 (56.7%)				
	Total	40 (100.0%)	22 (100.0%)	58 (100.0%)	120 (100.0%)	Fisher’s Exact Test (2-sided p<0.001)			
Panel D. EMS action item 3	No (0)	37 (92.5%)	19 (86.4%)	2 (3.4%)	58 (48.3%)	90.780	2	<0.001	0.870
	Yes (1)	3 (7.5%)	3 (13.6%)	56 (96.6%)	62 (51.7%)				
	Total	40 (100.0%)	22 (100.0%)	58 (100.0%)	120 (100.0%)	Fisher’s Exact Test (2-sided p<0.001)			

Percentages were calculated within ambulance-team categories. Panel A compares EMS arrival time (<10 versus ≥10 minutes); because only six respondents reported arrival within 10 minutes and expected-cell assumptions were not met, an exact test was used and Cramér’s V was not emphasized. Panels B-D assess reported performance of chest and abdominal examination (Q9.2), pulse measurement (Q9.3), and blood-pressure measurement (Q9.4), respectively, coded as 0 = No and 1 = Yes. Pearson’s chi-square assumptions were satisfied for Panels B-D, and Cramér’s V represents the magnitude of each respondent-level association, not a causal effect. Potential clustering within EMS units was not accounted for.

Reported arrival-time distributions differed across ambulance-team categories [Kruskal–Wallis H(2) = 13.747, *p* = .001; epsilon-squared=.100]. Respondents reporting 1–5 teams had a median arrival time of 10 min (IQR 10–15; mean 12.25 ± 2.52), compared with 10 min (IQR 10–10; mean 10.23 ± 1.07) among respondents reporting 6–15 teams and 10 min (IQR 10–10; mean 10.45 ± 2.82) among those reporting ≥16 teams. Bonferroni-adjusted Dunn comparisons identified differences between the 1–5-team category and both the 6–15-team category (adjusted *p* = .007) and the ≥16-team category (adjusted *p* = .003). The 6–15-team and ≥16-team categories did not differ (adjusted *p* = 1.000). Thus, the lowest team category had a less favorable reported arrival-time distribution, but the results did not demonstrate a progressive reduction in arrival time with each increase in team category (Table 5).

**Table 5:** Reported EMS arrival time according to ambulance-team category, including omnibus and pairwise rank-based comparisons (*n* = 120).

Section	Team category or comparison	*n*	Mean ± SD, min	Median (IQR), min	Range, min	Dunn z	Unadjusted p	Bonferroni-adjusted p
Panel A. Group distribution	1–5 teams	40	12.25 ± 2.52	10 (10–15)	10–15	-	-	-
	6–15 teams	22	10.23 ± 1.07	10 (10–10)	10–15	-	-	-
	≥16 teams	58	10.45 ± 2.82	10 (10–10)	5–15	-	-	-
Panel B. Dunn pairwise comparisons	1–5 vs. 6–15 teams	40 vs. 22	-	-	-	3.039	.002	.007
	1–5 vs. ≥16 teams	40 vs. 58	-	-	-	3.281	.001	.003
	6–15 vs. ≥16 teams	22 vs. 58	-	-	-	−0.529	.597	1.000

Kruskal–Wallis H(2) = 13.747, *p* = .001; epsilon-squared=.100. Pairwise comparisons were performed using Dunn's rank-sum procedure with Bonferroni correction. The arrival-time variable contained only four observed values: 5, 6, 10, and 15 min. The analyses are respondent-level and exploratory.

Reported minute-valued arrival time showed a modest inverse Spearman correlation with reported ambulance-team category (*ρ*=−0.277, *p* = .002; Supplementary Table S4). This item-level association was exploratory and did not account for traffic, distance, dispatch priority, workload, case severity, or possible clustering of personnel within EMS operational units.

## 4 Discussion

### 4.1 Knowledge-practice discrepancy among inpatient clinicians

Because the inpatient item blocks lacked adequate internal consistency and contained substantial response restriction, their findings were interpreted at the individual-item level, and no conclusions were based on composite scale scores. Cronbach's *α* was reported only as an internal-consistency diagnostic and was not interpreted as evidence of questionnaire validity (22, 24).

The paired findings indicate that discordance was concentrated in validated risk-score use and the specified airway-protection scenario, whereas the other clearly matched actions were reported frequently by almost all clinicians; current ACG and ESGE guidance recommends early Glasgow-Blatchford-based risk stratification in patients with UGIB (7, 9). The 33.3% discordance for risk-score use is clinically relevant because an international multicenter prospective study found that the Glasgow-Blatchford score performed best for identifying patients at very low risk of intervention or death (27). The airway result requires more nuanced interpretation because ESGE discourages routine prophylactic intubation and reserves it for selected patients with ongoing hematemesis and impaired airway control, while systematic-review evidence has not demonstrated a clear benefit from routine prophylactic intubation and has identified potential harms (7, 28). Because both endorsement and implementation were self-reported, these findings identify priorities for objective clinical audit but do not establish verified case-level non-adherence (29). At the individual-item level, these distributions suggest a discrepancy between endorsement of guideline-based recommendations and consistent self-reported implementation of selected actions. Because the practices were self-reported and measured cross-sectionally, these findings should not be interpreted as objectively observed nonadherence or attributed to clinician age, seniority, or clinical exposure (21, 25).

Our findings are consistent with surveys in other settings demonstrating that knowledge does not necessarily translate into practice. A cross-sectional study of emergency representatives in China reported an average knowledge score of 11.2 ± 3.5 out of 22; although most respondents recognized peptic ulcer disease as the leading cause of UGIB, only 52.7% identified endoscopy as the gold standard for diagnosis, and fewer than half correctly reported that endoscopy should occur within 24 h (30). Less than half of those representatives knew appropriate pharmacological therapy, and only 9.4% had the capacity to initiate transfusion within 10 min (30). In an Egyptian quasi-experimental study, only 8.57% of nurses demonstrated satisfactory practice in UGIB management before a training program; after intervention, knowledge and practice improved significantly (31). A large survey of emergency room nurses found that 91.43% had unsatisfactory practice, citing staff shortages and high patient volumes as major obstacles (31). These studies provide context for the observed difference between guideline endorsement and self-reported implementation; however, the present survey did not identify the mechanisms underlying that difference.

Finally, the absence of institutional audits or mandatory protocols may allow individual practice variation to persist. Recognizing these barriers is essential to designing interventions, such as simulation-based education, mandatory periodic training or digital decision support, that can bridge the knowledge-practice divide.

### 4.2 Adoption of risk stratification tools and attitudes toward guidelines

The second key finding concerns clinicians' attitudes toward risk scoring and their reported use of these tools. Although respondents almost universally endorsed guideline-based risk stratification, consistent implementation was less common. Clinicians with ≥5 years of experience more frequently considered UGIB risk scores clinically useful than clinicians with shorter tenure. This respondent-level association suggests that reported attitudes differed across tenure categories; however, the cross-sectional design and unmeasured institutional clustering preclude causal interpretation (25, 26). The present data do not permit reliable identification of the reasons underlying non-use of risk scores.

International guidelines unanimously recommend the use of validated risk scores, such as the Glasgow-Blatchford score (GBS) and Rockall score, to stratify patients, predict the need for intervention and identify those suitable for early discharge (9). The European Society of Gastrointestinal Endoscopy advises using the GBS at presentation and performing endoscopy within 24 h after stabilization (7). The American College of Gastroenterology supports similar thresholds and emphasizes that urgent (<6 h) endoscopy does not improve outcomes for most non-variceal bleeds (9). Despite these recommendations, adoption remains inconsistent. In a multicenter prospective study from France, only 12% of patients with suspected UGIB received any prehospital intervention, and formal risk stratification was rarely performed (13, 32). A recent review on scoring systems identified that while the GBS has excellent predictive value for endoscopic intervention and mortality, clinicians often prefer clinical judgement due to the perceived complexity of the score (12). Furthermore, a survey of trainees and trainers in the United Kingdom reported that fewer than one in three trainees felt competent in performing hemostatic endoscopy, and both groups emphasized the need for structured training and simulation-based courses (33). These studies support our observation that knowledge of risk scores does not ensure their use, and that educational and structural barriers must be addressed. Interventions aimed at improving adoption of risk stratification have shown efficacy. A one-day hemostasis course led to significant improvements in knowledge and procedural skills among general practitioners and surgical residents (34). Simulation cases for emergency medicine residents increased confidence in managing UGIB and emphasized key risk factors and the importance of timely endoscopy (35). A dedicated simulation exercise focusing on airway management during UGIB improved anesthesiology trainees' confidence in performing cricothyrotomy and emphasized mortality risk factors such as peptic ulcer disease, varices, NSAID use and alcohol (36). These interventions highlight the potential of targeted educational programs to enhance risk stratification adoption.

The discrepancy between positive attitudes toward risk scores and their limited use may reflect both cognitive and systemic factors. Clinicians may be unfamiliar with the specific variables and thresholds involved in risk scores or may lack readily accessible tools to calculate them during busy shifts. The fact that more experienced clinicians in our study cited lack of knowledge as the main barrier suggests that training in risk score use has not kept pace with guideline updates. Incorporating risk score calculators into electronic health records and mobile applications could mitigate this barrier. Another factor is the perception that clinical judgement suffices; while experienced clinicians often have good intuition, evidence suggests that structured scores more accurately predict need for intervention, particularly in borderline cases (12). Finally, in resource-limited settings, the practical implications of risk scores such as identifying patients who can be discharged early may be less relevant if outpatient follow-up is difficult to arrange. Tailoring risk stratification implementation to the local context and providing continuing education may therefore be critical to improving adoption.

### 4.3 Prehospital performance and resource allocation

The categorized arrival-time finding was based on only six respondents, all of whom were in the ≥16-team category; consequently, the exact-test result should be considered descriptive and does not provide strong evidence that greater team availability independently improves response time (25). The large Cramér's V values nevertheless indicate strong respondent-level associations between reported team category and the three clinical-assessment actions, although the cross-sectional design does not establish that increasing the number of teams would itself improve clinical behavior (25). Pulse and blood-pressure measurement are particularly relevant in suspected gastrointestinal bleeding because they permit calculation of the shock index, which has shown associations with mortality and hospital admission in prehospital gastrointestinal-bleeding data (37). Because EMS-unit identifiers were unavailable, responses from personnel working within the same operational unit may have been correlated, and the reported *p* values should therefore be interpreted as exploratory rather than as precise estimates of system-level differences (25).

The rank-based analysis of reported arrival-time values provided a more detailed assessment than the binary 10-minute threshold and showed a less favorable distribution in the 1–5-team category (38, 39). However, the absence of a difference between the 6–15-team and ≥16-team categories does not support a progressive or dose-dependent relationship between team availability and arrival time (38). EMS response time may also be influenced by dispatch demand, ambulance distance, traffic, geographic coverage, deployment practices, and case priority, several of which were not measured in the present survey (38, 39). Accordingly, the observed differences should remain explicitly exploratory and should not be described as demonstrating an independent effect of ambulance capacity (25).

Similar associations between resource allocation and response times have been reported elsewhere. A Japanese cohort found that response times shortened after ambulance resources were reallocated based on call volume and geographic need; the number of dispatches correlated strongly with response time extension (r = 0.94, *p* < 0.0001) (38). In the United States, EMS response times averaged 12.98 min in rural areas compared with 8.26 min in urban settings, with no significant association with median household income (40). These studies suggest that geography and resource distribution significantly influence prehospital timeliness. Our findings contribute exploratory respondent-level evidence regarding the association between team availability and arrival time in a Central Asian urban setting.

Prehospital triage tools may also improve outcome prediction and resource utilization. A retrospective study of EMS data in the United States found that a higher shock index (heart rate/systolic blood pressure) predicted mortality and hospital admission in patients with suspected GI bleeding (37). The authors proposed integrating the shock index into EMS protocols to identify high-risk patients and to prompt early hospital notification and mobilization of endoscopy teams (37). Prehospital guidelines from the Queensland Ambulance Service emphasize maintaining airway patency, administering supplemental oxygen, nil-by-mouth status, upright positioning and early hospital pre-notification for suspected variceal bleeding (15, 20). However, the same guidelines note that 85% of UGIB cases are non-variceal and that prehospital interventions are mostly supportive (20). These recommendations align with the practice patterns observed in our EMS data and underscore the need for basic training and equipment rather than advanced interventions in the prehospital phase. Educational and organizational interventions can enhance prehospital performance. Simulation training for EMS personnel has been shown to improve knowledge and confidence in managing gastrointestinal bleeds (35). Furthermore, creating dedicated call categories and dispatch protocols for UGIB may improve prioritization and resource deployment.

Resource constraints in EMS systems are a global challenge, particularly in low- and middle-income countries. Respondents reporting greater team availability also reported shorter arrival times and more frequent completion of selected assessments; however, these cross-sectional associations do not establish that increasing team numbers would produce these improvements. However, scaling up resources is often limited by financial and workforce considerations. Innovative solutions, such as optimizing dispatch algorithms, reallocating teams based on demand patterns, implementing community first responder programmes or expanding helicopter-based transport in remote areas (14), may provide incremental improvements. Implementing simple physiological triage tools like the shock index could further enhance prehospital decision-making and help align prehospital care with in-hospital resource availability.

### 4.4 Structural availability and protocol completeness

The final key result concerns institutional readiness. Most hospitals surveyed reported having written protocols for UGIB management, including policies for laboratory testing, blood transfusion thresholds and referral pathways. However, the existence of protocols did not guarantee practice adherence; clinicians' self-reported behavior often diverged from these guidelines. Rural hospitals noted longer transport times and limited access to around-the-clock endoscopy, reflecting national data showing that gastrointestinal bleeding remains a significant cause of surgical admission in Kazakhstan and that mortality is approximately 3%–4% (17). This finding addresses the study objective of analyzing organizational aspects of care and identifying opportunities to improve protocol implementation.

Discrepancies between protocol availability and actual practice are well documented. In a prospective observational study across multiple emergency departments, adherence to guidelines for UGIB management varied widely, with delays in endoscopy often attributed to organizational factors rather than clinical decision-making (13). A national UK audit reported that only 74% of hospitals had a formal bleeding management protocol, and even fewer audited adherence (33). In a training survey, gastroenterology trainees and their supervisors highlighted the lack of exposure to homeostatic procedures and insufficient simulation opportunities (33). These gaps mirror our findings and emphasize that guidelines must be accompanied by systems for training, auditing and feedback.

The presence of protocols is a necessary but insufficient condition for guideline adherence. Without enforcement mechanisms, regular audits and resource allocation, protocols may remain paper exercises. The present survey did not verify whether the reported resources and written protocols were consistently operational or familiar to frontline clinicians. Ensuring that protocols are integrated into routine workflows, perhaps through checklists or digital reminders, and that adherence is monitored, may enhance their effectiveness. Facility-level audit should verify out-of-hours endoscopy access, escalation pathways, interventional-radiology and surgical availability, and antiplatelet-management guidance. Collaboration between hospital leadership, emergency departments and endoscopy units will be essential to operationalize these changes.

### 4.5 Study limitations

This study has several limitations that warrant consideration. First, its cross-sectional design limits causal inference, and the associations observed between clinician characteristics and practice patterns cannot be interpreted as causal relationships. Second, the survey relied on self-reported data, which may be subject to recall bias and social desirability bias; clinicians might overestimate adherence to guidelines or underreport deviations. Moreover, although the questionnaires underwent multidisciplinary review and informal pretesting, they were not formally psychometrically validated; consequently, the findings should be interpreted at the individual-item level rather than as measurements of unified knowledge, attitude, or practice constructs (21, 22, 24). In particular, items focused on routine guideline-based work practices may have been too obvious or leading, which likely compressed the response range and contributed to the ceiling effect. This restricted variability affected internal-consistency estimates and did not support construction of composite scales. Third, the study involved respondents from four hospitals and one municipal EMS system in Almaty region, limiting generalizability to other regions of Kazakhstan or other countries with different healthcare structures. Fourth, we did not measure patient outcomes such as mortality, re-bleeding rates, transfusion requirements or length of hospital stay. Without these data, the clinical significance of the observed practice variations remains speculative. Fifth, responses from clinicians working within the same institution or EMS operational unit may have been correlated. Because the analyses treated respondents as independent, standard errors may have been underestimated and *p* values may be anticonservative; inferential findings should therefore be regarded as exploratory (26). Sixth, although we assessed the number of ambulance teams and calls per shift, other factors affecting response times such as traffic congestion, weather conditions, dispatch algorithms and paramedic fatigue were not captured.

### 4.6 Future directions

Future research should adopt longitudinal or interventional designs to assess the impact of organizational and educational interventions on clinical outcomes. Prospective cohort studies or cluster randomized trials could evaluate whether simulation-based training, risk-score calculators integrated into electronic health records or mandatory protocols improve practice adherence and reduce morbidity and mortality. Evaluating patient-centered outcomes would also allow estimation of the cost-effectiveness of such interventions. Expanding the survey to additional regions and incorporating rural and private hospitals would improve representativeness and allow comparison across healthcare systems. Integrating simple physiological scores such as the shock index into EMS protocols could be tested as a means of improving prehospital triage and aligning resource mobilization (37). Finally, qualitative research exploring clinicians' and paramedics' perceptions of barriers to guideline adherence would help tailor interventions to local contexts; such studies could utilize focus groups or in-depth interviews to capture nuanced insights. Implementation science frameworks could guide the design and evaluation of these interventions, ensuring sustainability and scalability.

## 5 Conclusion

This cross-sectional survey identified a mixed profile of gastrointestinal bleeding care in the Almaty region: hospital representatives consistently described structural endoscopy availability, but important elements of written protocol content were reported less consistently. Inpatient clinicians displayed high awareness of guideline-based UGIB management, yet key safety and triage practices especially consistent risk-score use and airway protection before endoscopy in high-risk presentations were not implemented uniformly. In the EMS sample, reported arrival time and completion of selected clinical assessments varied across categories of ambulance-team availability, although the small number of less-than-10-minute arrivals and unmeasured operational factors limit interpretation. Together, these respondent-level findings suggest that variation in GIB care may reflect both clinical-practice factors and differences in how services are staffed, structured, and coordinated across the emergency pathway. The findings support facility-level verification, objective clinical audit, and prospective evaluation of protocol implementation, clinician support, and EMS operations.

## Data Availability

The raw data supporting the conclusions of this article will be made available by the authors, without undue reservation.
