# Supplementary material for: Gastrointestinal bleeding care across hospital and EMS settings in the Almaty region: a cross-sectional survey of organization, practice, and service capacity
Source: Front Health Serv. 2026 Aug 18;6:1918526. doi: 10.3389/frhs.2026.1918526 (PMC13529710; doi:10.3389/frhs.2026.1918526)
Supplement: Supplementary file 1 [file Supplementaryfile1.docx]

Table S1. Internal-consistency diagnostics for questionnaire item blocks; no composite scales were retained.

| Scale | Items | Cronbach’s α | Standardized α | Items removed (zero variance) | Interpretation |
| --- | --- | --- | --- | --- | --- |
| Initial assessment (Q7a–Q7c and Q7e–Q7i) | 8 retained | Not computed | – | 7a, 7b, 7e, 7f, 7g, 7h, 7i | Insufficient variability; no composite retained. |
| Practice patterns (Q8a–Q8i) | 9 | -3.788 | 0.155 | 8d, 8g, 8h | Internal consistency not supported; no composite retained. |
| Attitudes/confidence (Q11a–Q11e) | 5 | 0.057 | 0.136 | 11b, 11c, 11e | Internal consistency not supported; no composite retained. |
| EMS clinical actions (Q9 block) | 5 | 0.741 | 0.602 | None | Acceptable (borderline) |

Table S2. Knowledge and guideline-based attitudes regarding initial management of upper gastrointestinal bleeding among inpatient clinicians (retained items Q7a–Q7c and Q7e–Q7i; n=99).

| Item | Strongly agree n (%) | Agree n (%) | Neutral/Disagree/Strongly disagree n (%) |
| --- | --- | --- | --- |
| 7a. Initial assessment should include CBC/electrolytes/blood typing/cross-match | 99 (100.0) | 0 (0.0) | 0 (0.0) |
| 7b. Initial assessment should include a validated risk score | 99 (100.0) | 0 (0.0) | 0 (0.0) |
| 7c. Orthostatic vital signs should be documented in hemodynamically stable patients | 64 (64.6) | 35 (35.4) | 0 (0.0) |
| 7e. Large-bore IV catheters should be placed during initial assessment | 99 (100.0) | 0 (0.0) | 0 (0.0) |
| 7f. Hypovolemic patients should receive crystalloid infusion during initial assessment | 99 (100.0) | 0 (0.0) | 0 (0.0) |
| 7g. Hypoxemic patients should receive supplemental oxygen during initial assessment | 99 (100.0) | 0 (0.0) | 0 (0.0) |
| 7h. Non-responders to fluid resuscitation should be admitted to ICU/monitored setting | 99 (100.0) | 0 (0.0) | 0 (0.0) |
| 7i. Airway protection before endoscopy in active hematemesis + altered mental status | 99 (100.0) | 0 (0.0) | 0 (0.0) |

Footnote: Q7d duplicated Q7c verbatim in the administered questionnaire. Its recorded distribution was 99/99 (100.0%) “strongly agree”; because it did not represent a distinct content item, it was excluded from substantive and psychometric analyses.

Table S3. Self-reported clinical practice during initial management of upper gastrointestinal bleeding among inpatient clinicians (Items 8a–8i, n=99).

| Item | Always n (%) | Often n (%) | Sometimes n (%) | Rarely/Never n (%) |
| --- | --- | --- | --- | --- |
| 8a. Initial labs are ordered (CBC/electrolytes/blood typing/cross-match) | 67 (67.7) | 32 (32.3) | 0 (0.0) | 0 (0.0) |
| 8b. Validated risk score is used in initial assessment | 1 (1.0) | 65 (65.7) | 33 (33.3) | 0 (0.0) |
| 8c. Orthostatic vital signs are documented | 66 (66.7) | 33 (33.3) | 0 (0.0) | 0 (0.0) |
| 8d. NG aspiration findings are documented (if NG tube present) | 99 (100.0) | 0 (0.0) | 0 (0.0) | 0 (0.0) |
| 8e. Large-bore IV catheters are placed | 0 (0.0) | 98 (99.0) | 1 (1.0) | 0 (0.0) |
| 8f. Hypovolemic patients receive crystalloid infusion | 1 (1.0) | 98 (99.0) | 0 (0.0) | 0 (0.0) |
| 8g. Hypoxemic patients receive supplemental oxygen | 99 (100.0) | 0 (0.0) | 0 (0.0) | 0 (0.0) |
| 8h. Non-responders to fluids are admitted to ICU/monitored setting | 0 (0.0) | 99 (100.0) | 0 (0.0) | 0 (0.0) |
| 8i. Airway protection before endoscopy (active hematemesis + altered mental status) | 0 (0.0) | 1 (1.0) | 98 (99.0) | 0 (0.0) |

Table S4. Exploratory item-level Spearman correlations in inpatient and EMS samples.

|  | Variable 1 | Variable 2 | Spearman’s rho (ρ) | P value | N |
| --- | --- | --- | --- | --- | --- |
| Inpatient | Clinical position | Frequency of managing UGIB | -0.478 | <0.001 | 99 |
| EMS | Arrival time after receiving the call (minutes) | Number of ambulance teams providing care simultaneously | -0.277 | 0.002 | 120 |

Footnote: Spearman’s rank correlation was used (two-tailed). Correlations are shown for inpatient and EMS respondents (Setting = 1 and 2). Negative coefficients reflect the direction of coding in SPSS (i.e., lower numeric codes correspond to more senior position / more frequent UGIB exposure / stronger agreement / “Yes” response). These respondent-level correlations were not adjusted for potential clustering within hospitals or EMS operational units and should be interpreted as exploratory.
